# Reaction of 2,6-dichloroquinone-4-chloroimide (Gibbs reagent) with permethrin – an optical sensor for rapid detection of permethrin in treated wood

**DOI:** 10.1186/1752-153X-7-122

**Published:** 2013-07-16

**Authors:** Mohamad Nasir Mat Arip, Lee Yook Heng, Musa Ahmad, Siti Aishah Hasbullah

**Affiliations:** 1 Forest Products Division, Forest Research Institute Malaysia, Selangor DE 52109, Malaysia; 2 Faculty of Science and Technology/South East Asia Disaster Prevention Research Institute (SEADPRI), Universiti Kebangsaan Malaysia, Bangi, Selangor DE 43600, Malaysia; 3 Faculty of Science and Technology, Universiti Sains Islam Malaysia (USIM), Nilai, Negeri Sembilan 71800, Malaysia

**Keywords:** 2,6-dichloro-p-benzoquinone-4-chloroimide, Permethrin, Nafion, Sol–gel, Optical sensors

## Abstract

**Background:**

A novel optical sensor for the rapid and direct determination of permethrin preservatives in treated wood was designed. The optical sensor was fabricated from the immobilisation of 2,6-dichloro-p-benzoquinone-4-chloroimide (Gibbs reagent) in nafion/sol–gel hybrid film and the mode of detection was based on absorption spectrophotometry. Physical entrapment was employed as a method of immobilisation.

**Results:**

The sensor gave a linear response range of permethrin between 2.56–383.00 μM with detection limit of 2.5 μM and demonstrated good repeatability with relative standard deviation (RSD) for 10 μM at 5.3%, 100 μM at 2.7%, and 200 μM at 1.8%. The response time of the sensor was 40 s with an optimum response at pH 11.

**Conclusions:**

The sensor was useful for rapid screening of wood or treated wood products before detailed analysis using tedious procedure is performed. The validation study of the optical sensor against standard method HPLC successfully showed that the permethrin sensor tended to overestimate the permethrin concentration determined.

## Background

Preservatives have been widely used in wood preservation process, agriculture, chemical, and polymer technology to protect various products against decay by biodegradation [1]. The choice of preservative to protect a product such as wood-based materials and vegetables is based on the chemical properties of the preservative [2]. Wood preservative usually consists of a mixture of preservatives. Wood preservative acts as an antifungal agent and insect repellent. In general, a preservative must have an appropriate level of toxicity to prevent spoilage from molds and to prevent insects from attacking wood or vegetable [3]. In the past, preservatives such as lindane, dieldrin, aldrin, and chlorpyrifos were widely used. Nowadays, these chemicals are largely replaced with pyrethroid group of preservatives such as permethrin and cypermethrin [4]. Permethrin is often used to protect wood from termite attack. The advantage of using this insecticide is that it is active in small doses and has a low toxicity to humans. Therefore, permethrin is used in solvent-based systems for the treatment of wood-based composites [5].

Usually, the quality of permethrin treatment in wood and vegetables is analysed using gas chromatography (GC), liquid chromatography (LC), immunology, and electronic nose [6-9]. These instrumental methods can normally determine permethrin concentrations in wood or vegetables according to the specifications set by standard procedures associated with the effective prevention of pest attack that causes biodegradation. Gas chromatography (GC) and liquid chromatography (LC) are techniques that cannot be used for *in situ* determination of permethrin. In addition, the sample for both techniques also requires an extraction step, which very often is time consuming. Other than that, the use of electronic noses could only detect permethrin qualitatively, i.e., whether it is present or absent in a sample.

**Figure 1:**
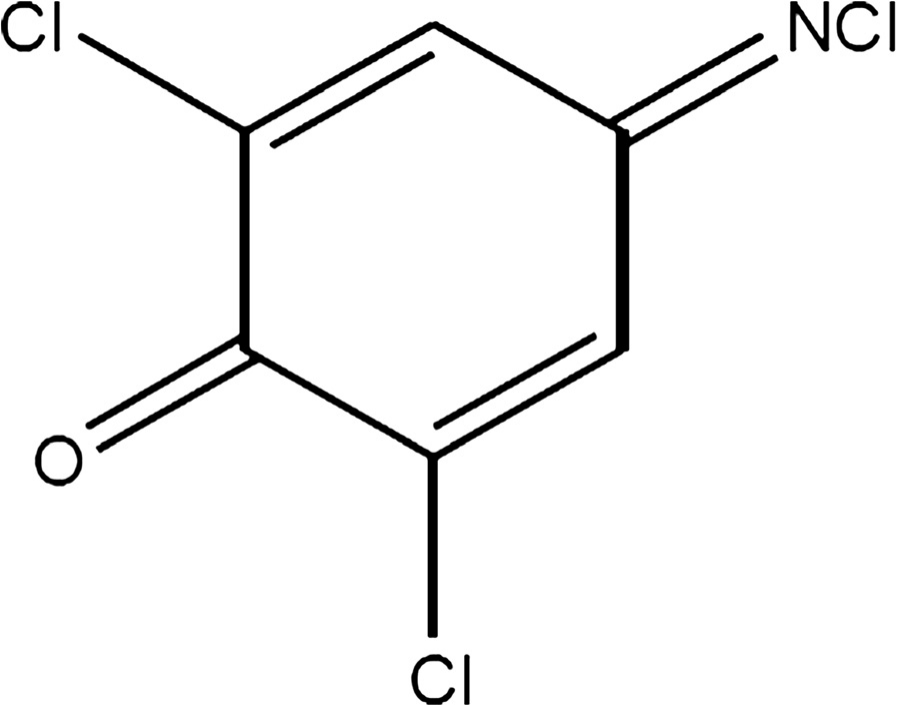
Structure of 2,6-dichloro-p-benzoquinone-4-chloroimide reagent.

In this study, an optical chemical sensor for the detection of permethrin in treated wood was developed. The new chemical sensor concept was based on the reaction between permethrin and 2,6-dichloro-p-benzoquinone-4-chloroimide reagent (Gibbs reagent). Gibbs method is a standard method used for the detection of phenol [10]. The method is based on the condensation reaction between dichloroquinone-4-chloroimide with phenol compounds that do not have a successor group to form a compound of the 2,6-dichlorophenol. The reaction takes place in an alkaline medium at pH 9.4 of borate buffer. For the determination of phenol in the range of ppm, 2,6-dichlorophenol compounds give absorption at a wavelength of 595 to 630 nm. In addition, such factors as temperature, pH, and presence of other compounds such as sulphide, reducing agent, and thiocresol have been found to affect the reaction. The structure of Gibbs reagent is shown in Figure 1. Until now, there is report regarding the reaction between permethrin and Gibbs reagent. An optical sensor is fabricated to detect permethrin by using 2,6-dichloro-p-benzoquinone-4-chloroimide reagent immobilised in a nafion and sol–gel silicate hybrid membrane. In this study, the performance of the chemical sensor for the analysis of permethrin in treated wood was validated with standard methods.

## Results

### Chemical reaction

**Figure 2:**
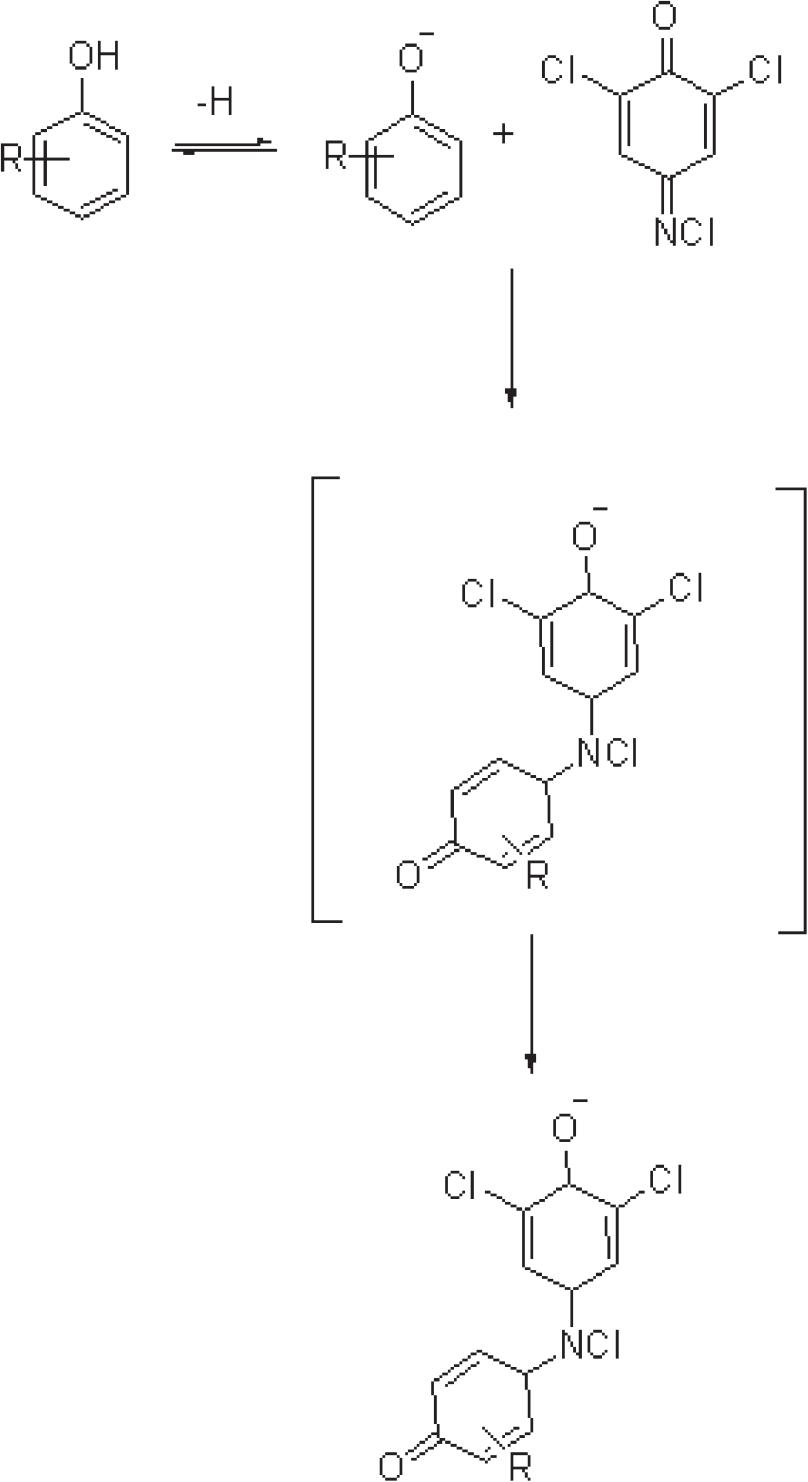
Scheme of the proposed mechanism for the reaction of Gibbs’ reagent with a phenolic residue of permethrin.

The chemical reactions between permethrin and Gibbs reagent are illustrated in Figure 2.

### UV–vis study

**Figure 3:**
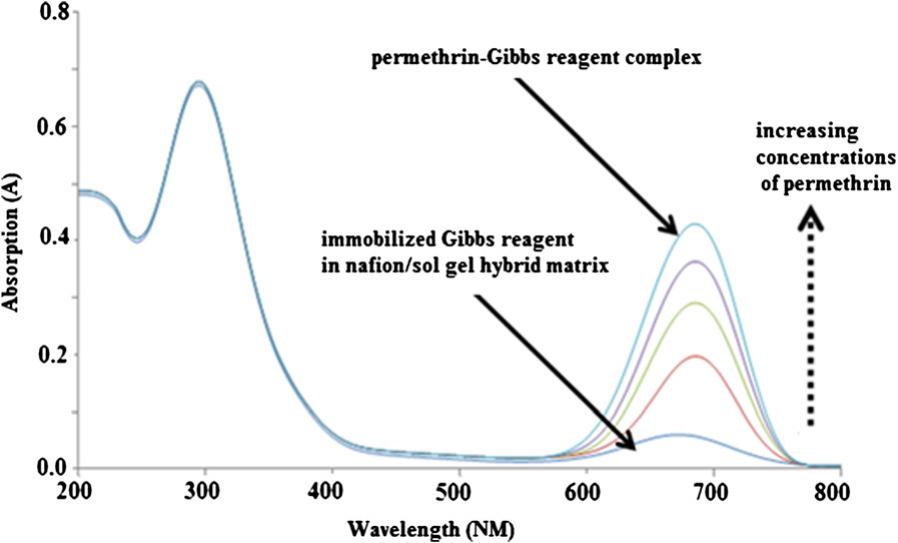
Gibbs reagent absorption spectrum of the absorbed film nafion/sol–gel with permethrin concentrations of 0.0–150.0 μM and pH 9.0.

The absorption spectrum of Gibbs reagent immobilised in the hybrid film nafion/sol–gel silicate is shown in Figure 3. As shown in the figure, the increase in absorption was due to the complex formation of permethrin-Gibbs when Gibbs reagent immobilised in the film reacted with permethrin where the yellow colour changed to blue colour.

### Effect of nafion/sol–gel ratio

**Figure 4:**
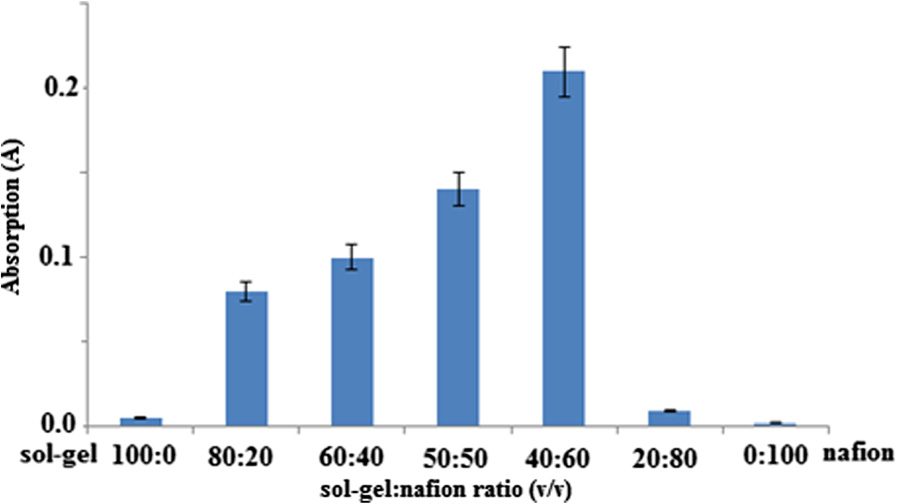
**Effects of the nafion/sol–gel silicate ratio towards chemical sensor response.** Permethrin concentration: 50.0 μM; concentration of Gibbs reagent: 1.0 M; pH: 9.0.

The effects of varying the ratio of nafion/sol–gel silicate on the chemical sensor response are shown in Figure 4.

### Effect of pH

**Figure 5:**
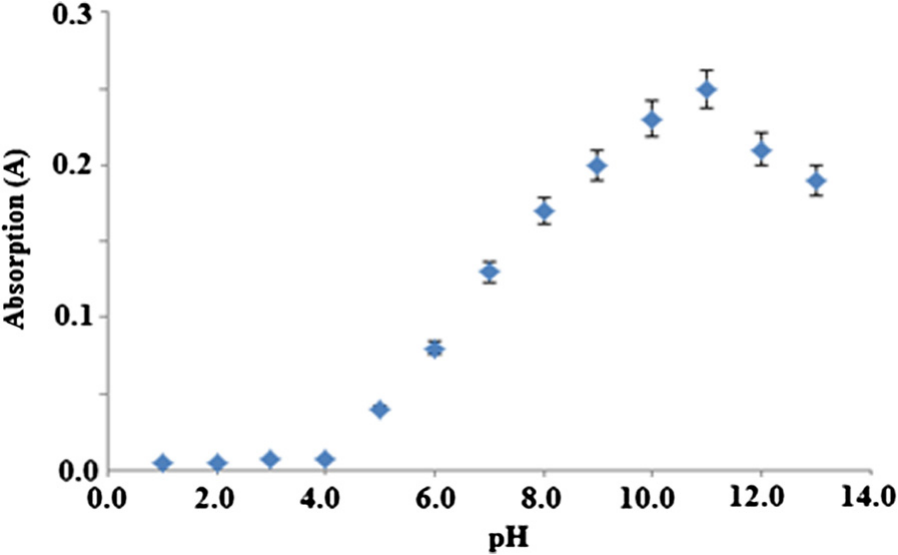
**Effects of pH on the chemical sensors response. **Permethrin concentration: 50.0 μM; concentration of Gibbs reagent: 1.0 M.

Figure 5 shows the effect of pH towards buffer solution in the pH ranging from 1.0 to14.0 on the chemical sensor response. Optimal chemical sensor response was found at pH 11.0.

### Effect of reagent concentration

**Figure 6:**
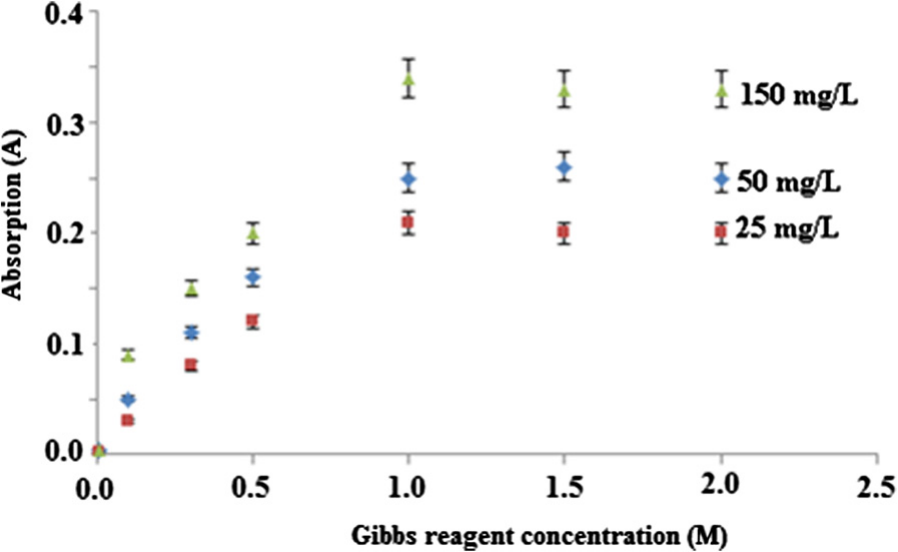
**Effects of concentration of Gibbs reagent on chemical sensor response. **Permethrin concentrations: 25.0, 50.0, and 100.0 μM; pH: 11.0.

The effect of Gibbs reagent loading on the permethrin-Gibbs complex formation was studied by measuring the intensity of the complex formed at a wavelength of 670 nm. Gibbs reagent concentrations studied were in the range of 0–2 M and permethrin concentrations used were 25 μM, 50 μM, and 100 μM in buffer solution at pH 11.0 (Figure 6).

### Leaching study

**Figure 7:**
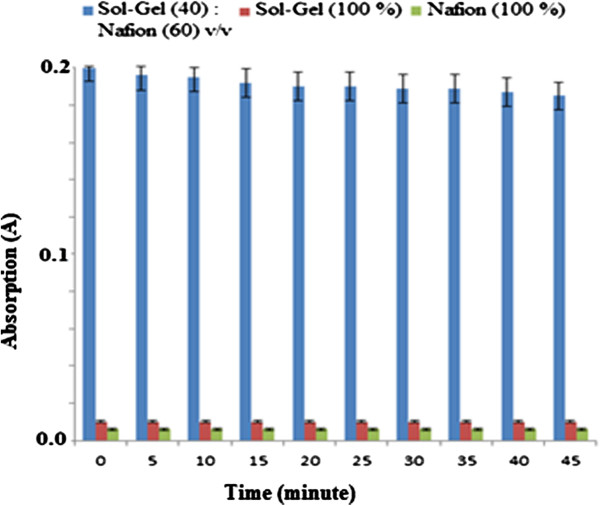
**Leaching study on chemical sensor response. **Permethrin concentration: 50.0 μM; concentration of Gibbs reagent: 1.0 M; pH: 11.0.

The effect of leaching of Gibbs reagent in response to optical sensors in buffer pH 11.0 containing permethrin for various immobilisation matrices is shown in Figure 7.

### Kinetic study

**Figure 8:**
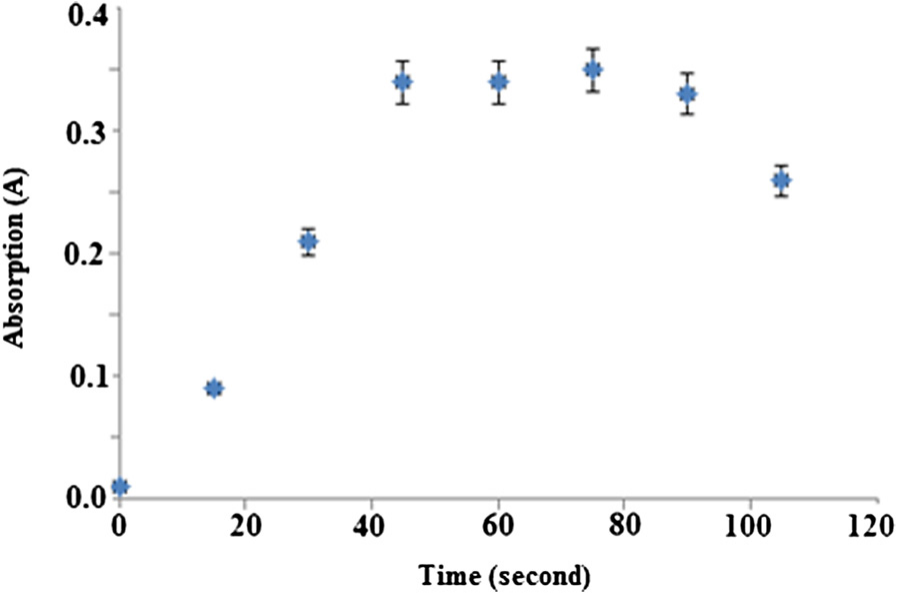
**Time response profile of the permethrin optical sensor. **Permethrin concentration: 100.0 μM; concentration of Gibbs reagent: 1.0 M; pH: 11.0.

Figure 8 shows the time taken by the optical sensor to respond to permethrin in concentration of 100 μM.

### Dynamic range

**Figure 9:**
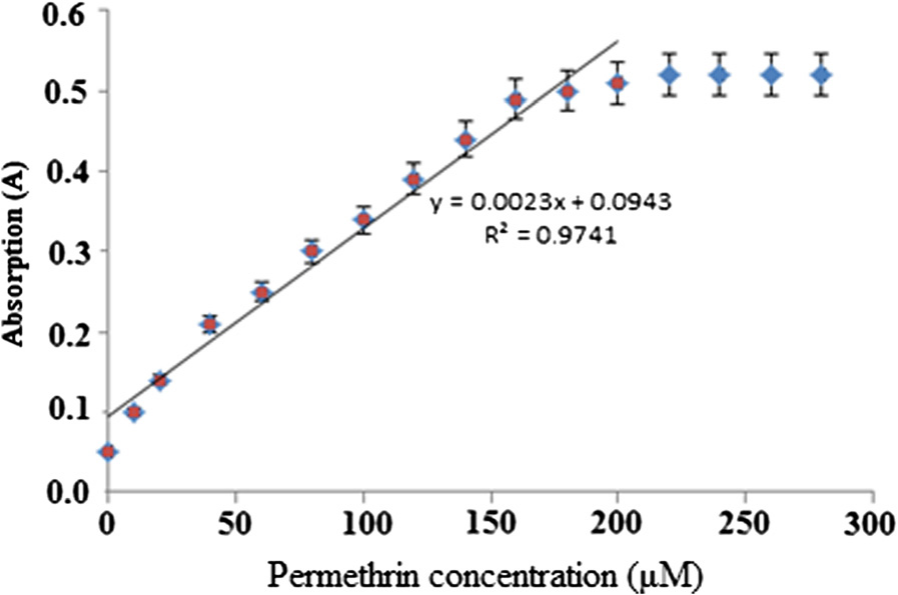
Dynamic range of concentrations of permethrin (0.0–300.0 μM) with Gibbs reagent 1.0 M at pH 11.0.

The linear response range of the optical sensor towards permethrin was 0–150 μM (R^2^ = 0.9900) (Figure 9). The value of the detection limit is 2.50 μM.

### Reproducibility study

**Figure 10:**
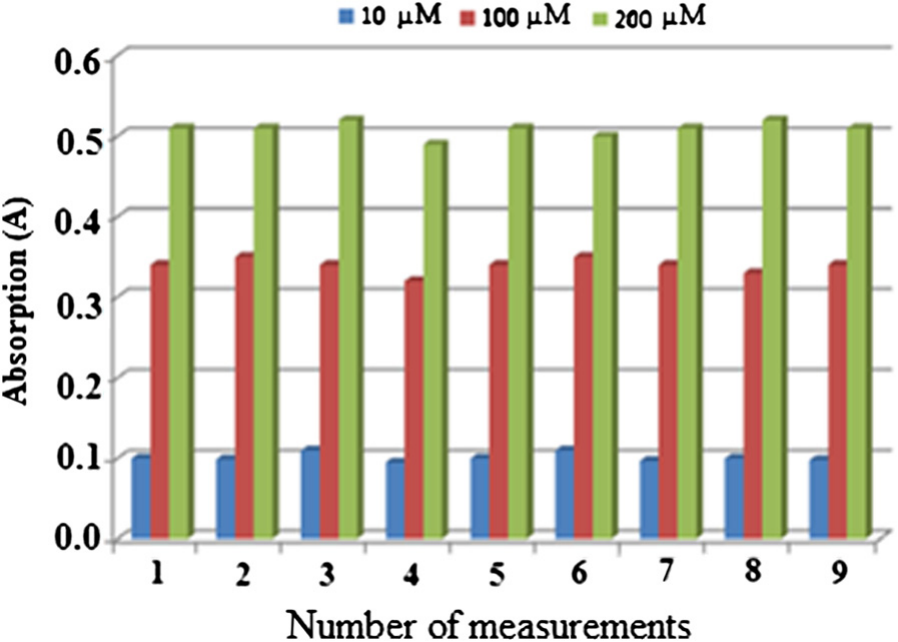
**Reproducibility study of permethrin optical sensor. **Permethrin concentrations: 10, 100, and 200 μM; concentration of Gibbs reagent: 1 M; pH: 11.0.

Reproducibility for Gibbs reagent immobilised in the nafion/sol–gel hybrid film refers to the measurement performed using different sensors of the same batch. Reproducibility study was performed at three different concentrations of permethrin, 10.0 μM, 100.0 μM, and 200.0 μM, as shown in Figure 10.

### Sensor lifetime study

**Figure 11:**
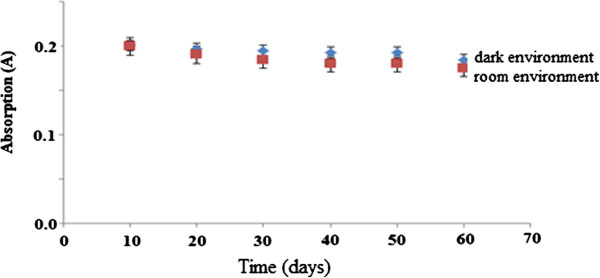
**The lifetime study of permethrin sensor response. **Permethrin concentration: 50.0 μM; concentration of Gibbs reagent: 1.0 M; pH: 11.0.

As shown in Figure 11, it appears that for the two-month study period (60 days), the permethrin optical sensors yielded RSD values of 4.86% and 2.76% respectively for dark and bright environments, indicating that the immobilised Gibbs reagent was stable for the study period of 60 days.

### Validation and recovery study

Validation study of the permethrin sensor was performed by comparing the analysis of treated wood spiked sample with permethrin using sensor and standard method such as HPLC (Table 1). The permethrin sensor developed in this study gave recovery values much higher than that of HPLC method, i.e., at the range of 120–130%.

**Table 1:** Recovery and precision values for permethrin determination in spiked samples and treated wood then analysed using developed chemical sensors and HPLC

**Method**	**Wood (spiked samples)**			**Treated wood**		
	**Type of sample**	**Recovery**	**Precision**	**Type of sample**	**Recovery**	**Precision**
			**RSD (%)**			**RSD (%)**
Chemical Sensors	Sample A^*^	130	5.2	Kempas^**^	126	6.2
	Sample B^*^	125	6.8			
	Sample C^*^	120	5.3			
	Sample A^*^	129	6.2	Rubberwood^***^	124	5.2
	Sample B^*^	123	7.0			
	Sample C^*^	122	5.2			
HPLC	Sample A^*^	95	4.8	Kempas^**^	98	3.5
	Sample B^*^	101	3.5			
	Sample C^*^	98	4.1			
	Sample A^*^	97	4.4	Rubberwood^***^	99	2.8
	Sample B^*^	105	4.3			
	Sample C^*^	94	3.9			

*Sample A = 10 μM; Sample B = 20 μM; Sample C = 50 μM.

**Kempas was treated with 0.02% w/w of permethrin preservative. The treatment was done using vacuum impregnation vessel.

***Rubberwood was treated with 0.02% w/w of permethrin preservative. The treatment was done using vacuum impregnation vessel.

## Discussion

In this study, tetraethyl orthosilicate (TEOS) was used as a starting material for the preparation of sol–gel silicate for the immobilisation of Gibbs reagent. It is known that the material properties of sol–gel silicate matrix, such as surface area, pore size and distribution, are influenced by many factors during the preparation of the sol–gel silicate including pH and ratio of silica to water. Nafion is a polymer that has hydrophobic backbone fluorocarbon, while sol–gel, which is a cation converter, is characteristically hydrophilic; thus, nafion/sol–gel shows medium hydrophobic character [11]. This property helps the chemical sensor to retain the reagent dye in the film and in reducing leaching. Therefore, in this study, nafion mixed with sol–gel silicates to form organic–inorganic hybrid material was used to immobilise the Gibbs reagent. In addition, the nature of this hybrid material can overcome the cracking problem commonly experienced by the sol–gel film of pure silicate [11-13].

Gibbs reagent (2,6-dichloro-p-benzoquinone-4-chloroimide) has long been used for detecting phenol and its derivatives [14]. In this study, the degradation of permethrin in ethanol under alkaline condition was assumed to form a phenolic residue that can be detected by the Gibbs reagent. The mechanism is likely to involve an oxidative coupling reaction to generate a p-quinoid species. A free parahydroxyl from the phenyl ring is used to initiate a dehydrogenative reaction. The reaction yields a 2,6-dichloroindophenol compound (dye complex) that gives a blue colour.

The absorption spectrum of Gibbs reagent immobilised in the hybrid film nafion/sol–gel silicate is shown in Figure 3. The absorption increase was due to the complex formation of permethrin-Gibbs when the Gibbs reagent immobilised in the film reacted with permethrin, turning the yellow colour to a blue colour.

Hydrophobic hybrid and high porosity material were to immobilise the hydrophilic Gibbs reagent and to prevent leaching of soluble chemical from the sensor film. The effects of varying the ratio of nafion/sol–gel silicate on the chemical sensor response are shown in Figure 4. Lower optical response was observed when pure silicate sol–gel matrix material was used for the immobilisation of Gibbs reagent. This behaviour may be due to the nature of the hydrophilic silicate film that could not withstand excessive Gibbs reagent loaded into the matrix material [15]. Hybrid material with a ratio of 40%:60% (v/v) sol–gel and nafion showed optimal response. The increase in nafion content, which was higher than 60% (v) in the matrix film, reduced the intensity of the optical sensor response. The decrease in sensor response when the nafion content was higher than 60% was due to the reduction of porosity of silicate sol–gel film with the increase in nafion content in the hybrid material and with the increase in the hydrophobic properties. Therefore, the decrease in sensor response affected the amount of Gibbs reagent immobilised in nafion/sol gel silicate hybrid network. When the hybrid material porosity decreased, the amount of immobilised reagents Gibbs also reduced. The porosity reduction in hybrid chitosan/sol–gel silicate caused the amount of immobilised horseradish peroxidase enzyme to reduce thus leading to poor sensor response [13]. The reduction in chemical sensor response could also be due to increase in hydrophobic properties of the hybrid material as the permethrin became difficult to diffuse into sensor membrane containing Gibbs reagent. As a result, weak response was obtained. The film thickness of chemical sensor was calculated based on weight of coated layer of nafion/sol–gel silicate immobilised with Gibbs reagent. The film thickness for nafion/sol–gel silicate in the ratio 40:60 (v/v) was estimated in the range of 4–5 μm.

Next, Figure 5 shows the effect of pH towards buffer solution in the pH range from 1.0 to 14.0 on the chemical sensor response. Optimal chemical sensor response was found at pH 11.0. Therefore, the buffer at pH 11.0 was selected for use in further studies. This is similar to the results reported by Palacio (1979) in his analysis of the colorimetric method of determination of capsaicin in using vanadium oxytrichloride [16]. An increase in the permethrin concentration increases the formation of permethrin-Gibbs complex and results in an absorption increase. The pH of the reaction plays an important role in the complex formation. As mentioned earlier, it is postulated that under alkaline conditions, permethrin will decompose to yield a phenolic residue, which will couple with Gibbs reagent. This coupling reaction is highly dependent on pH because it will determine the pace of the formation of 2,6-dichloroquinoneimine as active species for the formation indophenols (blue product) after reacting with phenolic compounds. As stated by Svobodova et al. (1978) in their study on the reaction of Gibbs reagent and phenol in solution, Gibbs reagent decomposition to the formation of 2,6-dichloroquinoneimine occurs within the pH range of 7.5–10.0 [17]. The rate of decomposition of Gibbs reagent increases as the pH increases (pH 7.5–10.0). At pH 6–7, quinoneimine formation is very slow. Coupling reaction or the formation of 2,6-dichloroquinoneimine occurs fastest in alkaline medium with an optimum pH range of 8–10 (Siggia & Hanna 1979; Svobodova et al. 1978) [17,18]. In alkaline conditions (>pH 7.5), phenol functional groups will experience deprotonation to form nucleophilic, anionic phenoxide (C_6_H_5_O^-^) groups that are highly water soluble and a strong director (strong activator) that will determine the outcome of the reaction that occurs at the ortho or para position (McMurry 2008) [19]. Phenol in the form of phenoxide anion will then react with 2,6-dichloroquinoneimine and yield 2,6-dichloroindophenol compound (dye complex) to give a blue colour. Thus, the coupling reaction between Gibbs reagent and phenol (or other phenolic compounds) occurs most rapidly in alkaline medium.

In this study, the effect of Gibbs reagent loading on the permethrin-Gibbs complex formation was studied by measuring the intensity of the complex formed at a wavelength of 670 nm. Gibbs reagent concentrations studied were in the range of 0–2 M and permethrin concentrations used were 25 μM, 50 μM, and 100 μM in buffer solution at pH 11.0. At all permethrin concentrations, the intensity of the absorption of permethrin-reagent complex reached maximum level at the Gibb reagent concentration of 1.0 M, as shown in Figure 6. Thus, this concentration of 1.0 M Gibbs reagent was used as a condition for determination of permethrin using the chemical sensor.

The effect of leaching of Gibbs reagent in response to optical sensors in buffer pH 11.0 containing permethrin for various immobilisation matrices is shown in Figure 7. From immersion time of 0–5 min, Gibbs reagent leaching was 1%, 80%, and 90% respectively for nafion/sol–gel silicate, pure silicate sol–gel, and pure nafion. The composition of 40% sol–gel and 60% nafion demonstrated almost no leaching of sensor components. This was because under this optimal mixture, there was a suitable hydrophobicity phase in the film to prevent leaching.

Next, Figure 8 shows the time taken by the optical sensor to respond to permethrin in concentration of 100 μM. The response time was fast for an optical sensor, which was about 40 s to reach steady-state response. This shows that properties of reagent do not change when the Gibbs reagent is immobilised in nafion/sol–gel hybrid matrix. The linear response range of the optical sensor was of 0–150 μM of permethrin (R^2^ = 0.9900) (Figure 9). The value of the detection limit is 2.50 μM. This response range was somewhat lower than that of permethrin using non-immobilised Gibbs reagent (2.56–383.00 μM) as a result of the more restricted movement of permethrin through the hybrid polymeric matrix compared to reaction at the liquid phase.

Reproducibility of Gibbs reagent immobilised in the hybrid film nafion/sol–gel refers to the measurement performed using different sensors of the same batch. In this study, reproducibility study was performed at three different concentrations of permethrin namely 10.0 μM, 100.0 μM, and 200.0 μM, as shown in Figure 10. However, the repeatability study could not be done because the sensor could not be reused or regenerated. The RSD values for the fabrication of optical permethrin sensors were 5.3% (n = 10), 2.7% (n = 10), and 1.8% (n = 10) respectively for 10.0 μM, 100.0 μM, and 200.0 μM. According to Alabbas (1989), variations of the sensor response are caused by two factors, fabrication and operation of the sensor [20]. These variations include the variations caused by the quantity and particle size sensor matrix that is then linked to variations produced by the immobilised reagent concentration on support material (transducer). However, in this study, the main reason causing the poor response was more focused on sensor fabrication.

The sensor lifetime study was performed under two different conditions namely bright and dark conditions at room temperature for a specified period of time. Two conditions were chosen to investigate any differences that might exist. As shown in Figure 11, for the two-month study period (60 days), the permethrin optical sensors yielded RSD values of 4.86% and 2.76% respectively for dark and bright conditions, indicating that the immobilised Gibbs reagent was stable for the study period of 60 days.

Validation study of the permethrin sensor was performed by comparing the analysis of treated wood spiked sample with permethrin using sensor and standard method such as HPLC. The permethrin sensor developed in this study gave recovery values much higher than that of HPLC method, i.e., at the range of 120–130%. The RSD values under precision study for both method were <10% (n = 10). Statistical analysis of the data showed that there were significant differences between the two methods of determining permethrin. These were the results of the less selective nature of the permethrin sensor as it was found to respond slightly to the wood extracts such as sugar and starch even in the absence of permethrin.

## Conclusion

An optical chemical sensor using of 2,6-dichloro-p-benzoquinone-4-chloroimide immobilised on nafion/sol–gel silicate film for the determination of permethrin in treated wood has been developed. The response of the optical sensor to permethrin was linear with response time of 40 s. The optical sensor for permerthrin showed sensitivity, good reproducibility, and stability. However, validation study of the optical sensor against standard method HPLC showed significant difference between the two methods; the permethrin sensor tended to overestimate the permethrin concentration. This may be due to the slightly lack of selectivity of the sensor towards permethrin. We suggest that the sensor can be applied for rapid screening of wood or treated wood products before detailed analysis using tedious procedure is performed.

## Methods

### Reagents and solutions

All chemicals used were of analytical grade. Throughout the study, deionised water was used for solution preparation. Permethrin standard was purchased from Merck (Darmstadt, Germany). Permethrin stock solution (1200 μM) was prepared by dissolving 0.3 g permethrin powder in ethanol (99%) and diluted to 250 mL. Gibbs reagent was obtained from Fluka. Gibbs stock solution (9.5 × 10^-2^ M) was prepared by dissolving 1 g of the reagent in ethanol and diluted to 50 mL. Buffer solutions were prepared according to the methods from Handbook of Basis Tables for Chemical Analysis (Svoronos 1989) [21].

### Apparatus

Calibrated Perkin-Elmer, Model Lamda 35 Ultraviolet–visible Spectrophotometer was used. A calibrated Shimadzu HPLC, model SPD-M10AVP with PDA detector, column used Geminibrand Phenomenex, flow rate 1.5 mL/min was used for validation. All glassware was calibrated according to the MS ISO/IEC 17025 requirement.

### Procedure for HPLC analysis

Before the sample solution was injected into the HPLC, extraction solution was injected into the HPLC sample bottle using a syringe containing a nylon membrane, which was used to protect the head from damage. The volume of the sample was 20 μL. Sample bottles were then arranged in a rack and the HPLC analysis was then carried out automatically. The HPLC instrument used was Shimadzu HPLC, column type: Phenomenex Luna^®^, 5 μm Silica(2) 100 Å, LC Column 100 × 4.6 mm in room temperature. Ultraviolet detector was set at a wavelength of 260 nm. For the wood samples, the mixture of n-hexane and tetrahydrofuran (THF) (95:5, v/v) was used as mobile phase.

### Measurement of the absorption spectrum

The absorption spectrum of chemical sensors based on the Gibbs reagent immobilisation of layered hybrid film nafion/sol–gel silicate in the presence of permethrin (100.0 μM) in buffer solution at pH 9.0 was recorded using a UV–vis spectrophotometer. For the effect of permethrin concentration on the sensor response, the concentrations of permethrin were between 0.0 μM and 150 μM. Absorption spectra for the sensor were recorded at wavelengths of 300–800 nm at an interval of 1 min for 5 min.

### Procedure for evaluation of various parameters on optimum permethrin sensor response

For the purposes of assessing the effect of the nafion/sol–gel silicate, the ratios of 100:0, 80:20, 60:40, 50:50, 40:60, 20:80, and 0:100 (v/v) were used and the film chemical sensor was included. Nafion/sol–gel silicate hybrid solutions were prepared by mixing nafion (5% solution in a mixture of alcohol) and sol–gel silicate solution in an airtight bottle. The nafion volume ratios of sol–gel silicate used were 100:0, 80:20, 60:40, 50:50, 40:60, 20:80, and 0:100 (v/v). The mixture was stirred to produce a homogeneous solution and then left overnight at room temperature before use. Gibbs reagent concentration used was set at 2 M, pH 9.0, and permethrin concentration of 100.0 μM. Chemical sensor response was recorded at the wavelength of 670 nm for 5 min.

The effect of pH in response to chemical sensors in the pH range of 1.0–14.0 was studied. Permethrin concentrations were determined at a concentration of 100.0 μM. Gibbs reagent concentration used was set at 2 M. Chemical sensor response was recorded at the wavelength of 670 nm for 5 min.

In this study, the estimated load stationary Gibbs reagent in the hybrid film nafion/sol–gel silicate used was in the range of 0 M to 2 M. Permethrin concentration used was 100 μM in buffer solution pH optimum. Chemical sensor response was recorded at a wavelength of 670 nm for 5 min. Gibbs reagent leaching effects were studied by soaking the chemical sensor in buffer solution pH optimum for 0, 5, 15, 25, and 40 min before the evaluation of the performance of the chemical sensor response was performed using 100.0 μM permethrin. Chemical sensor response was recorded at the wavelength of 670 nm at an interval of 1 min for 5 min.

### Analytical performance of permethrin chemical sensor

#### Sensor response, reproducibility, and repeatability study

After the optimisation process was done, the optimal value was used in the study of dynamic range of concentrations of permethrin. Permethrin concentration range studied was between 0.0 μM to 500.0 μM. pH and concentration of the reagent were at the optimum level. Limit value detection (LOD) was also determined from the study of this dynamic range. For the analysis of chemical sensor reproducibility, chemical sensor immersed in a solution of concentrated permethrin 10.0, 100.0, and 200.0 μM and the absorption reading were recorded after 5 min. The same procedure was repeated using different chemical sensor film. After the result from the reproducibility analysis of the chemical sensors was known, repeatability study was done by dipping the chemical sensor into the permethrin solution with the concentrations of 10.0, 100.0, and 200.0 μM and the absorption was recorded after 5 min. Chemical sensor was then regenerated using a buffer solution at pH 2.0 before being dipped into permethrin solution with the same concentration. These steps were repeated until the readings for 10 measurements were obtained.

### Lifetime study

Lifetime study was performed to study the stability of this chemical sensor when stored for 90 days. The study was conducted using two different conditions: 1) chemical sensor stored in a volumetric flask wrapped in aluminium foil and placed in the refrigerator; and 2) chemical sensor stored in a volumetric flask without being wrapped in aluminium foil and placed at room temperature. The study was done using permethrin solution with concentration 50.0 μM. Absorption graph of concentration against time was plotted. Stability analysis of the chemical sensor was done after the chemical was kept for 3 months in the conditions specified above. Chemical sensor to be stored in desiccator was prepared. Dessicator was used to keep the chemical sensor dry and its stability was determined each week. For each measurement, three replicates of chemical sensors were used. Permethrin concentration was set at 100.0 μM. Chemical sensor response was recorded at the wavelength of 670 nm for 5 min.

### Validation and recovery study

In this study, the concentration of permethrin used was 50.0 μM. Then, permethrin solution was mixed with Kempas (*Koompassia malaccensis*) wood powder and Rubberwood (*Hevea brasiliensis*) powder. Readings from the optical sensor developed were taken. Then, readings from optical chemical sensor for the wood extracts containing permethrin were taken. In addition, the optical chemical sensor developed was used to test the material preserved from the same group of pyrethroid such as cypermethrin. Initially, only the readings for the reaction between optical chemical sensor were taken. After that, the permethrin chemical sensor was added into the mixture of permethrin and cypermethrin in the concentration ratio of 1:1. The same procedure was applied to other interference chemicals such as sugar and deltamethrin.

Recoveries of permethrin study sample were done by using wood samples. Sensors were calibrated with a solution of permethrin. Next, known weight of wood samples was crushed and added to permethrin solution of known concentration. Wood samples were also added to the buffer as control. Chemical sensor was then exposed to the wood sample. The permethrin response value added was determined using the equation,

x=y-z

where *x* is a real response of permethrin concentration added to the wood samples, *y* is the measured response of wood samples, and *z* is the measured response of wood samples before permethrin was added. By extending the slope of the line on the graph to cut the x-axis and y-axis, the actual concentrations of permethrin in wood samples can be determined by xM, while yM is the actual concentration of permethrin added to wood samples. Percent recovery can be calculated using the following formula,

$$\frac{x}{y}\times 100=\%$$

To determine that the developed chemical sensor showed same performance in terms of its analysis with the standard method, the performance of the chemical sensor was compared with that of Australian/New Zealand Standard. [22]. In this study, 1 g of wood powder was weighed and transferred into a 50 mL Erlenmeyer flask. Then, 15–20 mL of n-hexane was inserted into the flask. Next, the flask was covered with aluminium sheets and placed on an electric sieve. Wood powder immersed in the n-hexane was filtered for 30 min at a speed of 100 revolutions per minute. After 30 min, the wood powder was separated from the solvent extraction results through screening process by using size 4 filter paper. The extraction yield was then put into a 25 mL volumetric flask. N-hexane solution was added to the extraction yield up to level 25 mL. Thus, the permethrin analyte extraction from wood samples was available for analysis.

## Competing interests

All authors declare that they have no competing interests.

## Authors’ contributions

MNMA planned and carried out the sensor manufacturing, measurements, and data production. LYH headed the scientific planning and evaluation of the project. MA and SAH provided scientific advices. All authors read and approved the final manuscript.
